# Infection of the Ex Vivo Tonsil Model by HTLV-1 Envelope-Pseudotyped Viruses

**DOI:** 10.3390/pathogens12020182

**Published:** 2023-01-24

**Authors:** Mélanie Langlois, Salim Bounou, Michel J. Tremblay, Benoit Barbeau

**Affiliations:** 1 Axe des Maladies Infectieuses et Immunitaires, Centre de Recherche du Centre Hospitalier, Universitaire de Québec-Université Laval, Québec, QC G1V 4G2, Canada; 2 Euromed Research Center, Faculty of Pharmacy, Université EUROMED de Fès, Fez 30000, Morocco; 3 Département de Microbiologie-Infectiologie et Immunologie, Faculté de Médecine, Université Laval, Québec, QC G1V 0A6, Canada; 4 Département de Sciences Biologiques, Université du Québec à Montréal, Montréal, QC H3C 3P8, Canada; 5 Réseau Intersectoriel de Recherche en Santé de l’Université du Québec (RISUQ), Montréal, QC H2X 1E3, Canada

**Keywords:** HTLV-1, ex vivo tonsil model, pseudotyped viruses, tropism

## Abstract

Human T-cell leukemia virus type 1 (HTLV-1) is the causal agent of adult T-cell leukemia/lymphoma and HTLV-1-associated myelopathy/tropical spastic paraparesis. Its tropism is known to be broad in cultured cell lines, while in vivo data support a more selective transmission toward CD4+ T cells and the limited targeting of other hematopoietic cell types. An essential condition for HTLV-1 infection is cell-to-cell contact, to which both virological synapse and viral biofilm have been suggested to strongly contribute. As cell lines and animal models each present their own limitations in studying HTLV-1 replication, we have explored the use of an ex vivo model based on the secondary lymphoid tonsillar tissue. HIV-1 luciferase-expressing pseudotyped viruses bearing the HTLV-1 envelope protein at their surface were first shown to recapitulate the wide spectrum of infectivity of HTLV-1 toward various cell lines. Tonsil fragments were next exposed to pseudotyped viruses and shown to be reproducibly infected. Infection by HTLV-1 Env-pseudotyped viruses was blocked by different anti-gp46 antibodies, unlike infection by HIV-1 virions. The dose-dependent infection revealed a gradual increase in luciferase activity, which was again sensitive to anti-gp46 antibodies. Overall, these results suggest that the ex vivo tonsil model represents a reliable alternative for studying HTLV-1 replication and potentially viral latency, as well as early clonal formation.

## 1. Introduction

The human T-cell leukemia virus type 1 (HTLV-1) was the first retrovirus to be isolated in humans [1,2,3,4] and is estimated to currently infect from 5 to 10 million individuals worldwide [5]. HTLV-1 is the etiological agent of adult T-cell leukemia/lymphoma (ATL) and of HTLV-1-associated myelopathy/tropical spastic paraparesis (HAM/TSP), in addition to other less prevalent disorders that have been associated with this virus [6]. Patients diagnosed with ATL have a mean survival time of six months after diagnosis, regardless of the chosen treatment (such as chemotherapy, α-interferon/azidothymidine, and allogeneic bone marrow transplantation) [7,8].

HTLV-1 has a large tropism in vitro but has been shown to be more restricted to CD4+ T cells in vivo, although previous studies had provided evidence of the presence of proviral DNA in other hematopoietic (CD8+ T lymphocytes, monocytes, dendritic cells, B lymphocytes) as well as non-hematopoietic cells [9,10,11,12]. Despite this broad variability in cell tropism, cell-free HTLV-1 infection is very inefficient. Indeed, numerous studies have demonstrated that the transmission of HTLV-1 occurs mostly through cell-to-cell contact [13]. Three mechanisms have been proposed to account for this required cell-to-cell contact: the virological synapse [14], the viral biofilm [15], and the nanotube-based cellular conduit [13,16]. Aside from extensive studies over HTLV-1 infection conducted in cell culture, most infection experiments have relied on in vivo models, such as mouse, rabbit, and simian species. The use of such models remains tedious and they are not fully representative of the human environment, although humanized mouse models have shown successful infection and demonstrated ATL-like diseases [17,18,19,20].

Alternative cell culture models have been proposed for studying viral infection. These include organoid tissues and cultured tissue fragments, often referred to as tissue explants [21,22,23]. The ex vivo tonsil model has been extensively used for HIV-1-related studies and shown to be representative of secondary lymphoid tissues [24,25]. This tissue contains various cell types, such as dendritic cells (DCs), and T and B lymphocytes, and represents an environment facilitating intercellular interaction. In addition, several studies have shown that HTLV-1-infected T cells are present in lymphoid tissue, such as lymph nodes, and provide an environment for clonal evolution [26,27]. Interestingly, it has been proposed that tonsil tissue might be an important point of entry of HTLV-1 during breastfeeding, similarly to what has been reported for HIV-1 and SIV [28,29,30].

In this study, we have thereby examined the susceptibility of tonsil tissue toward HTLV-1 infection. Using HTLV-1 Envelope-pseudotyped viruses, we demonstrate the reproducible and specific permissiveness of this ex vivo model to infection. These results provide evidence that the ex vivo tonsil model could represent an alternative model to study HTLV-1 replication in lymphoid tissue and clonal evolution.

## 2. Materials and Methods

Cell lines. HEK293T (human embryonic kidney) and HOS-CXCR4 (human osteosarcoma) cells were grown in DMEM medium supplemented with 10% FBS and antibiotics (1% penicillin-streptomycin). The human Jurkat E6.1 T cell line was maintained in RPMI medium containing 10% FBS and antibiotics.

Plasmids and antibodies. The plasmid pNL4-3.Luc.E-R+ expresses the luciferase gene and is defective for Envelope protein synthesis (NIH HIV Reagent Program, Rockville MD) [31]. pHXB-Luc is another HIV-1-based reporter construct expressing the luciferase reporter gene and producing infectious viruses [32]. The plasmid pSV HTLV-1 env (kindly provided by Dr. R. Sutton, Baylor College of Medicine, Houston, TX, USA) and pCAGHTLV-1 env (obtained from Dr. K. Okuma, Kyushu University, Kukuoka, Japan) both express the HTLV-1 *env* gene under the SV40 and CMV promoter, respectively [33,34]. Anti-gp46 antibodies used in this study included the rat monoclonal antibody LAT-27 (kindly provided by Dr. Y. Tanaka, University of the Ryukyus, Okinawa, Japan) [35] and monoclonal antibodies SP2,3/4A (rabbit) and 0.5α (human) [36,37], both of which were provided by the NIH HIV Reagent Program and are known to be neutralizing.

Production of pseudotyped particles. HEK293T cells (4 × 10^5^) were transfected with a reporter viral DNA construct (7 μg) in the presence or absence of an *env* expression vector (3 μg) using the calcium phosphate protocol (CalPhos, Clontech laboratories, Palo Alto CA). At 48 h post-transfection, supernatants were harvested, passed through a 0.22 μm filter and stored at −80 °C until further use. The quantification of the viral production was conducted using a home-made p24 ELISA assay, as previously described [38].

Ex vivo tonsil model. Human tonsils were provided by the department of otorhinolaryngology of the Centre Hospitalier de l’Université Laval (CHUL, Quebec City, QC, Canada) from routine tonsillectomy within 4 h of excision. The tonsils were first soaked and rinsed in PBS containing antibiotics for 45 min and then cut into thin 2 to 3 mm^2^ blocks. Tissues (two fragments) were then overlaid on each collagen sponge gel (Pharmacia and Upjohn Inc., Kalamazoo, MI, USA), presoaked in supplemented RPMI medium in 6-well plates. A volume of 300 μL of additional supplemented medium was then added to allow tonsil sections to be at the air-liquid interface. Plates were incubated at 37 °C for 24 h before infection.

Infection of cell lines and the ex vivo tonsil model. HTLV-1 Envelope-pseudotyped viral particles and HXB-Luc viruses (20 ng p24, unless otherwise specified) were added to HEK293T, HOS-CXCR4, or Jurkat E6.1 cells in 96-well plates, which were then incubated for 24 h (or up to 96 h) at 37 °C. After the removal of half of the volume, cells were lysed in lysis buffer (0,4 mM DTT, 0.2% Triton-X100, 2% glycerol, 5 mM Tris phosphate pH 7.8) and incubated for 20 min. For tonsil fragments, various amounts of viruses were added on top of each fragment and then incubated for 72 h. Next, tissues were retrieved from the sponge gels and individually added to 50 μL of medium to which 5X lysis buffer was added, followed by the mincing of tissues. In certain infection experiments, viral samples were preincubated with anti-gp46 antibodies for 5 min at 37 °C. Luciferase activity was measured in a MLX Microtiter plate reader (Dynex Technologies, Chantilly, VA, USA), using luciferin as the substrate, as previously described [38]. All statistical analyses were carried out using Student’s t-test and differences were considered as significant with a *p* value lower than 0.05.

## 3. Results

The tonsil tissue represents a second lymphoid organ, which can be used as a model for lymph nodes. We were thus interested in assessing if HTLV-1 could be studied in this environment through a previously published protocol [38].

### 3.1. Testing of HTLV-1 Envelope-Pseudotyped Viruses 

**Figure 1:**
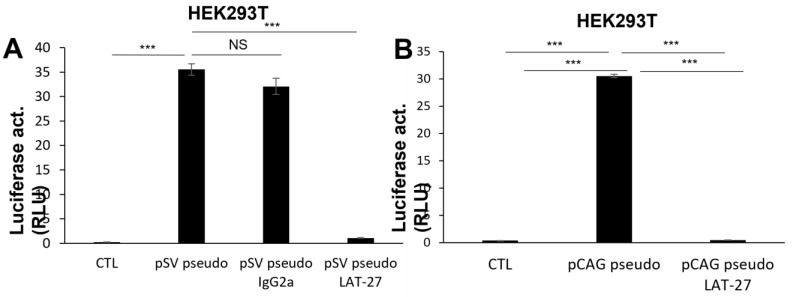

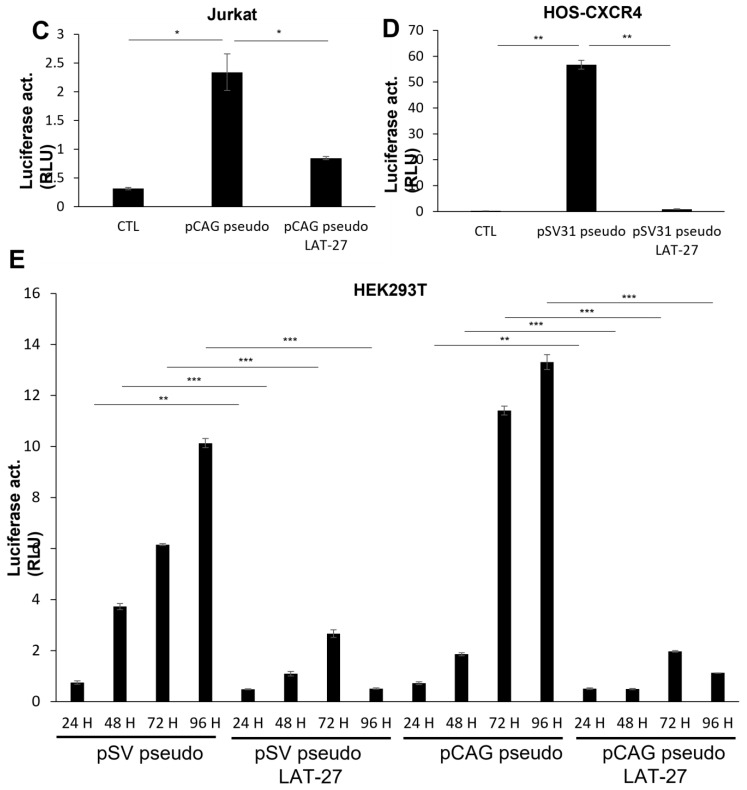
Infection of different cell lines by HTLV-1 Envelope-pseudotyped viruses. NL4-3.Luc-based virions pseudotyped with the HTLV-1 Envelope (20 ng p24) produced from HEK293T cells co-transfected with pCAG- or pSV-based expression vectors were first preincubated with anti-gp46 (LAT-27, 50 μg/mL) or non-specific IgG2a (50 μg/mL) antibodies (vs. no antibodies) for 5 min at 37 °C and then added to HEK293T (**A**,**B**,**E**), Jurkat (**C**), or HOS-CXCR4 (**D**) cells. After 24 h, cells were lysed and measured for luciferase activity. In panel **E**, infection was monitored at different time points postinfection (24 to 96 h). Each condition was performed in triplicates and results are presented as mean ± S.D. * *p* < 0.05, ** *p* < 0.01 and *** *p* < 0.001 (NS = non-significant). These data are representative of two independent experiments. CTL: no added viruses.

We first produced luciferase-expressing defective HIV-1 particles pseudotyped with the HTLV-1 Envelope proteins through the transfection of two different HTLV-1 *env* expression vectors (pCAG- and pSV-based) in HEK293T cells. The produced virions were tested in different cell types, such as HEK293T, Jurkat E6.1, and HOS-CXCR4, and were shown to lead to significant luciferase activity, which was sensitive to blocking LAT-27 antibodies, but not to non-specific IgG antibodies (Figure 1A–D). A time course experiment revealed an increase in signal from 24 to 96 h post-infection in HEK293T cells (Figure 1E). Anti-gp46 antibodies preincubated with the virus again blocked the infection, as indicated at the different time points.

Results thus demonstrated that both HTLV-1 Env-pseudotyped HIV viruses were infectious toward different cell lines and specifically blocked by anti-gp46 antibodies.

### 3.2. Infection of Ex Vivo Tonsil Model

**Figure 2:**
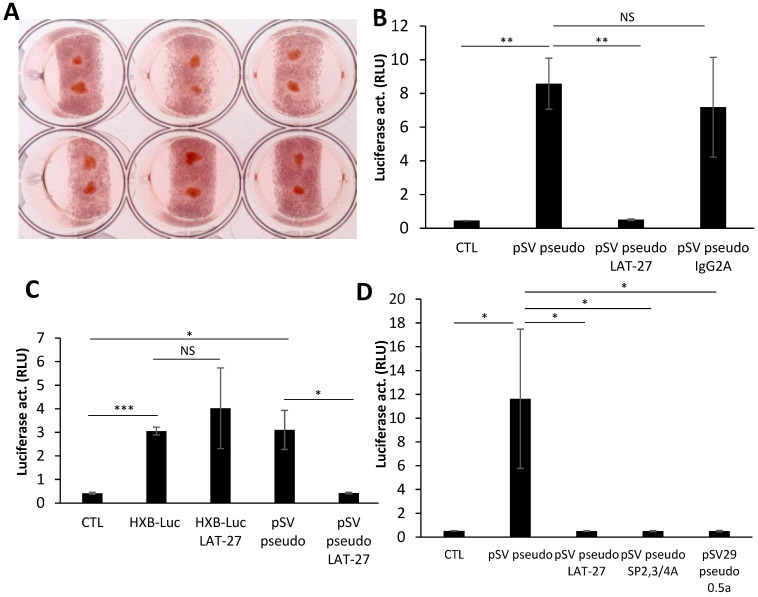
Infection of tonsillar sections by HTLV-1 Envelope-pseudotyped viruses. (**A**) Tonsil blocks were prepared as described in Materials and Methods and overlaid on sponge gels (two per gel) in a 6-well plate. (**B**–**D**) NL4-3.Luc-based virions pseudotyped with the HTLV-1 Envelope (1–3 ng p24) were preincubated with anti-gp46 (LAT-27, 50 μg/mL; SP2,3/4A, 1:200, and 0.5α, 1:100; panel (**D**)) or non-specific IgG2a (50 μg/mL) antibodies (vs. no antibodies) for 5 min at 37 °C and then added to tonsil fragments. In panel (**C**), infection of tonsil tissues with HXB-Luc virions was also performed in parallel. After 72 h, tissues were lysed and measured for luciferase activity. Each condition was performed in quadruplicates and results are presented as mean ± S.D. * *p* < 0.05, ** *p* < 0.01 and *** *p* < 0.001 (NS = non-significant). These data are representative of two independent experiments. CTL: no added viruses.

**Figure 3:**
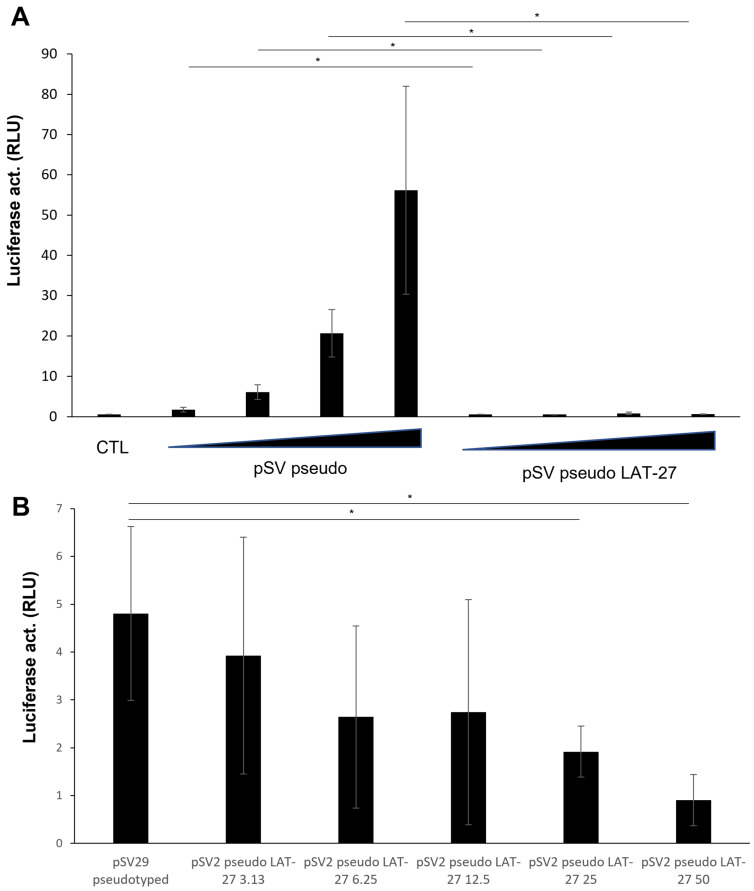
Dose-dependent infection of tonsil fragments and sensitivity to anti-gp46 antibodies. Tonsil blocks were infected by NL4-3.Luc-based virions pseudotyped with the HTLV-1 Envelope preincubated in the absence or presence of LAT-27 antibodies. In panel (**A**), increasing amounts of virions (0.5 to 4 ng p24) were added to tonsil blocks. In panel (**B**), different concentrations of LAT-27 antibodies (3.13 to 50 μg/mL) were tested in the preincubation step. After 72 h, tissues were lysed and measured for luciferase activity. Each condition was performed in quadruplicates and results are presented as mean ± S.D. * *p* < 0.05 (NS = non-significant). Data are representative of two independent experiments. CTL: no added viruses.

Tonsil fragments were prepared and cultured (Figure 2A), as described in the Materials and Methods section. Fragments were typically infected with 1 to 3 ng HTLV Env (SV-based)-pseudotyped viruses in the absence or presence of blocking LAT-27 antibodies. As depicted in Figure 2B, the infection of the tested tonsil fragments was confirmed and shown to be specific (LAT-27 vs. non-specific IgG2). The selective effect of LAT-27 toward HTLV-1 Env-pseudotyped viruses was further evaluated by comparing infection with HIV-1 luciferase-expressing viruses, which were not affected by anti-gp46 antibodies, while infection by HTLV-1 Env-pseudotyped viruses was again blocked (Figure 2C). Similar results were obtained when human tonsil fragments were infected with HIV-1-pseudotyped with pCAG-based HTLV-1 Env, but no signals above background levels were measured in infection experiments with non-Envelope-pseudotyped viruses (data not shown). Notably, variations between experiments in terms of signals were noted, but were reminiscent of intrinsic variations of tonsil preservation from different donors. To further confirm the specificity of the infection by HTLV-1 Env-pseudotyped viruses, other known anti-gp46 antibodies were similarly tested by the preincubation of the virus before the addition to tonsil tissues and again led to an important reduction in luciferase activity (Figure 2D). We further analyzed the infection of the ex vivo tonsil model by conducting a dose response experiment (increasing number of viral particles). As shown in Figure 3A, a gradual increase in luciferase activity was demonstrated in infected tonsil fragments, which were blocked when preincubated with LAT-27. We also tested different dilutions of LAT-27 and, as expected, infectivity was observed to be proportional to the antibody levels (Figure 3B).

These results thereby argue that tonsil fragments could be infected by HTLV-1 and therefore could represent a reliable model to study HTLV-1 replication/infection and feature characteristics of infected cells.

## 4. Discussion

The understanding of HTLV-1 infection remains important. Initial studies have pointed to differences in the tropism of cell lines in culture vs. in vivo [9,10,11,12]. Despite these differences, the low infectivity of cell-free HTLV-1 is a constant feature of this virus and highlights the importance of cell-to-cell contact for virus transmission. In particular, the virological synapse and virological biofilm have both been illustrated as two major mechanisms that intervene in HTLV-1 infection through these cellular contacts [13,16].

As cultured cells are generally ill-suited for studying these types of interactions, we have opted to use tonsil tissue as a representative model of secondary lymphoid tissue to address its susceptibility to HTLV-1 infection. Our choice was guided by the multicellular constitution of this tissue, which includes major cell targets and virus-transferring cells (CD4+ and CD8+ T cells and DCs) [39]. We thus made use of pseudotyped HIV-1 viruses bearing HTLV-1 Envelope on their surface to evaluate the potential susceptibility of tonsils to HTLV-1. Our first analyses were focussed on the infection of different cell lines by this pseudotyped virus, which was indeed confirmed. Importantly, anti-gp46 antibodies were also demonstrating the specificity of the signal.

The infection of tonsil tissue by pseudotyped viruses was performed next. Our results clearly showed infection, which was dependent on gp46, as shown by the blocking capacity of different anti-gp46 antibodies. Furthermore, HIV-1 HXB-Luc infection was not impacted by these antibodies, again demonstrating specificity. We were however limited in the amount of pseudotyped particles to be added, due to the maximal volume that can be reliably added on top of each tonsil block. Concentrated viruses would have improved the signal. As another limitation, we were not successful in initiating infection with cell-free infectious, replication-competent viruses (data not shown), although this might be expected, given the strict reliance on cell-to-cell contact for infection. 

Although mouse and simian models have obvious advantages for studying HTLV-1 in terms of replication and pathogenesis, they remain challenging. The ex vivo tonsil model offers several advantages and has actually been used for HIV-1 and other viruses [24,40,41,42,43]. For HTLV-1, the infection would strongly benefit from intercellular contact in this environment and could allow the virus to propagate in multiple CD4+ T cells in a timewise fashion. Interestingly, one of the important receptors for the HTLV-1 entry, neuropilin-1, is expressed in a subpopulation of T cells in tonsil tissue [44], which could therefore be a major target. Another advantage of tonsil fragments is the capacity to maintain blocks in culture for several days; it would then be interesting to monitor viral replication through time and to potentially identify cell clones with viral latency. Although we do not expect to be capable of studying cell transformation in this short time frame, we could nonetheless conduct a phenotypic assessment of CD4+ and CD8+ T cell populations and analyse cell proliferation in proviral DNA-positive cells vs. non-infected cells. Furthermore, transcriptomic analyses would be an informative approach to examine changes at the cellular level following infection and possibly early latency. We are however aware that this model does not recapitulate all the complexity occurring in an infected individual, such as the immune response and its targeting of viral replicating CD4+ T cells. In addition, we are aware that the use of pseudotyped viruses offers a more limited understanding of HTLV-1 replication in tonsil tissues, because cell entry, despite being crucial, is the sole replication step that is being represented. This again underscores the importance of conducting infection experiments with fully replicative HTLV-1 viruses. We are hence currently working on the infection of tonsil tissues through the addition of fixed HTLV-1-producing cells to improve HTLV-1 infection.

In conclusion, our infection experiments of tonsil tissue with HTLV-1 Envelope-pseudotyped viruses suggest that this ex vivo model provides an adequate environment to study HTLV-1 infection, although we are aware that infection experiments with fully replicating HTLV-1 viruses will be required to confirm its use. We are nonetheless confident that the future optimization of this model will allow us to study HTLV-1 infection in lymphoid tissues and address important questions in terms of the early generation of clones and their characteristics.

## Data Availability

Not applicable.
